# Metabolic engineering of medium-chain fatty acid biosynthesis in *Nicotiana benthamiana* plant leaf lipids

**DOI:** 10.3389/fpls.2015.00164

**Published:** 2015-03-24

**Authors:** Kyle B. Reynolds, Matthew C. Taylor, Xue-Rong Zhou, Thomas Vanhercke, Craig C. Wood, Christopher L. Blanchard, Surinder P. Singh, James R. Petrie

**Affiliations:** ^1^Commonwealth Scientific and Industrial Research Organization, Food and Nutrition FlagshipActon, ACT, Australia; ^2^NSW Department of Primary Industries, Graham Centre for Agricultural Innovation, Charles Sturt UniversityWagga Wagga, NSW, Australia; ^3^ARC Industrial Transformation Training Centre for Functional Grains, Charles Sturt UniversityWagga Wagga, NSW, Australia

**Keywords:** plant biomass, triacylglycerol, WRINKLED, Nicotiana, medium chain, fatty acid

## Abstract

Various research groups are investigating the production of oil in non-seed biomass such as leaves. Recently, high levels of oil accumulation have been achieved in plant biomass using a combination of biotechnological approaches which also resulted in significant changes to the fatty acid composition of the leaf oil. In this study, we were interested to determine whether medium-chain fatty acids (MCFA) could be accumulated in leaf oil. MCFA are an ideal feedstock for biodiesel and a range of oleochemical products including lubricants, coatings, and detergents. In this study, we explore the synthesis, accumulation, and glycerolipid head-group distribution of MCFA in leaves of *Nicotiana benthamiana* after transient transgenic expression of C12:0-, C14:0-, and C16:0-ACP thioesterase genes. We demonstrate that the production of these MCFA in leaf is increased by the co-expression of the WRINKLED1 (WRI1) transcription factor, with the lysophosphatidic acid acyltransferase (LPAAT) from *Cocos nucifera* being required for the assembly of tri-MCFA TAG species. We also demonstrate that the newly-produced MCFA are incorporated into the triacylglycerol of leaves in which WRI1 + diacylglycerol acyltransferase1 (DGAT1) genes are co-expressed for increased oil accumulation.

## Introduction

Vegetable oils, in the form of triacylglycerol (TAG), are an important global commodity with over 150 million tonnes being produced every year (FAO, 2003), which has remained relatively constant over recent years. Vegetable oils are an essential resource for various industries including, human and animal nutrition, the chemical industry and renewable energy. Due to demand for these plant oils, it is predicted that by 2020 worldwide production of vegetable oil is required to increase by 30% (Thoenes, 2011). This increase in production is likely to come in part from increases in arable land at the expense of endangered habitats.

The use of petroleum is widely accepted as being unsustainable. Therefore, it is necessary to develop alternative and sustainable resources to supplement or replace the extensive use of petroleum. In an attempt to expand global vegetative oil production, which currently relies on oilseeds and palm oil, various laboratories are now investigating the production of oil in non-seed biomass such as leaves. A variety of metabolic engineering approaches have resulted in increased biomass oil (Vanhercke et al., 2014b; Wood, 2014) including reducing TAG and fatty acid turnover (Slocombe et al., 2009), ectopic expression of transcription factors that regulate processes of seed development and maturation (Santos Mendoza et al., 2005), down-regulation of metabolic pathways that compete for available carbon (Sanjaya et al., 2011), and the over-expression of acetyl-CoA carboxyalse (ACCase) (Klaus et al., 2004). Furthermore, disruption of SUGAR-DEPENDANT1 (SDP1) in *Arabidopsis thaliana*, a lipase related pathway, also resulted in increased TAG accumulation in non-seed tissues (Kelly et al., 2013). Recently, much higher levels of oil accumulation in plant biomass was achieved using a combination of biotechnological approaches (Vanhercke et al., 2014a). This accumulation to 15% TAG in *Nicotiana tabacum* leaves was achieved by the coordinated transgenic expression of the WRINKLED1 transcription factor (WRI1), diacylglycerol acyltransferase (DGAT1) and oleosin genes. This breakthrough in leaf oil accumulation was ascribed to the synergistic increase in both fatty acid synthesis and oil synthesis (via WRI1 and DGAT1, respectively) (Vanhercke et al., 2013) and the formation of stabilized oil bodies (via oleosin). Not only was oil accumulated in leaves, the composition of the leaf oil was dramatically altered, including a reduction in α-linolenic acid in favor of oleic acid.

An important component of global oil consumption is medium-chain fatty acids (MCFA), namely fatty acids with less than 18 carbons, including those with 12, 14, and 16 carbons in length. These MCFA are ideal sources of biodiesel and also for a range of oleochemical feedstocks including, coatings, and detergents. The biochemical pathways for the production of MCFA have largely been elucidated. In plants, *de novo* fatty acid biosynthesis occurs in the plastid stroma, with the plastidial elongation of acyl chains being terminated by the activity of acyl-acyl carrier protein (acyl-ACP) thioesterases (Tjellstrom et al., 2013). These enzymes are therefore considered to be major determinants of fatty acid chain lengths, and hence the overall fatty acid profile of plants. However, it has been demonstrated that acyl-ACP synthetases can reactivate released fatty acids, hence allowing further elongation (Koo et al., 2005). Acyl-ACP thioesterases are typically classed as being either FatA or FatB, which are responsible for the release of oleic acid (C18:1^Δ9^), stearic acid (C18:0), palmitic acid (C16:0) and the MCFA, which in this study principally refers to lauric acid (C12:0), myristic acid (C14:0), and palmitic acid (C16:0) (Dörmann et al., 2000). Following export from the plastid, these fatty acids can be esterified to a glycerophosphate backbone via the Kennedy pathway in the endoplasmic reticulum to form TAG. Plant biotechnologists have investigated the accumulation of C12:0 and C14:0 in seed and leaf tissues of transgenic *Brassica napus* by the transgenic constitutive expression of a C12:0-ACP thioesterase from California Bay Laurel (*Umbellularia californica*) (Eccleston et al., 1996). This study demonstrated that significant levels of C12:0 could be accumulated in mature *B. napus* seeds. However, very low levels of C12:0 were observed in leaf tissue, despite high levels of C12:0-ACP thioesterase expression and *in vitro* activity. Similar results were obtained when the same gene was transformed in *A. thaliana* (Voelker et al., 1992). This research was extended by the co-expression of the *Cocos nucifera* lysophosphatidic acid acyltransferase (LPAAT) and thioesterase which resulted in an increased accumulation of total C12:0 as well as an increased fraction of trilaurin in the seeds of *B. napus* (Knutzon et al., 1999). Therefore, it can be observed that the expression of thioesterases and acyltransferases, enzymes involved in the biosynthesis of fatty acids and TAG, can be used to increase the accumulation of specific fatty acids, and hence tailor the overall fatty acid profile of plants.

Here, we explored whether MFCA production is compatible with high oil production in leafy biomass. We used a combinational expression platform to combine WRI1 and DGAT1 with various thioesterases and oil assembly enzymes to produce MCFA in leaf oil. A variety of thioesterases were found to drive the production of 12-, 14-, and 16-carbon MCFA in the leaves of *Nicotiana benthamiana*. The co-expression of AtWRI1 in particular increased the levels of leaf oil containing MCFA. Further improvements in MCFA accumulation of WRI1-dependent leaf oils was attained by using the CnLPAAT. High resolution LC/MS techniques were used to trace the accumulation of MCFA from their site of synthesis to their eventual deposition into oil. The biochemical pathways for the accumulation of MCFA in biomass oils and implications of production in stably-transformed crops are discussed.

## Materials and methods

### Construct assembly

Thioesterase protein sequences were used to synthesize codon optimized gene coding sequences (Geneart, Regensburg, Germany) for *Cinnamomum camphora* C14:0-ACP thioesterase [referred to as “Cinca-TE,” Q39473.1, (Yuan et al., 1995)], *Cuphea lanceolata* acyl-ACP thioesterase type B [Cupla-TE, CAB60830.1 (Töpfer et al., 1995)], *Umbellularia californica* C12:0-ACP thioesterase [Umbca-TE, Q41635.1, (Voelker et al., 1992)], *Cocos nucifera* acyl-ACP thioesterase FatB1 (Cocnu-TE1, AEM72519.1), *Cocos nucifera* acyl-ACP thioesterase FatB2 (Cocnu-TE2, AEM72520.1), *Cocos nucifera* acyl-ACP thioesterase FatB3 (Cocnu-TE3, AEM72521.1), and *Cuphea viscosissima* FatB1 (Cupvi-TE, AEM72522.1) all described by Jing et al. (2011). The *C. nucifera* LPAAT [CnLPAAT, Q42670.1, (Knutzon et al., 1995)] was also synthesized. Each gene was then cloned into the *Eco*RI site of a binary vector, pJP3343, which already contained a 35S promoter with duplicated enhancer region (Vanhercke et al., 2013). AtWRI1 and AtDGAT1 expression vectors were produced as previously described by Vanhercke et al. (2013). *Agrobacterium tumefaciens* strain AGL1 was transformed with each of the constructs.

### *N. benthamiana* transient assay

Transient expression in *N. benthamiana* leaves was performed as previously described (Wood et al., 2009), with some minor modifications. *A. tumefaciens* strain AGL1 harboring each binary vector (p19 viral suppressor protein and the chimeric gene of interest) was grown at 28°C in LB broth supplemented with the appropriate antibiotics. Cultures were centrifuged and gently resuspended in two volumes of infiltration buffer (5 mM 4–morpholineethanesulfonic acid (MES), 5 mM MgSO4, pH 5.7, 100 μM acetosyringone) and grown for a further 3 h. The optical densities of each culture were measured and adjusted to a final OD_600_ equal to 0.1 prior to infiltrations. The final mixture of AGL1 cells was infiltrated via syringe into the underside of leaves of 5 week old *N. benthamiana* plants (Voinnet et al., 2003). Infiltrated areas of leaves, commonly ~3 cm in diameter, were circled by a permanent marker. Infiltration experiments were conducted in quadruplicate using different leaves of similar age. The samples being compared were randomly located, with a p19 control infiltrated for each plant. After infiltrations, the *N. benthamiana* plants were grown for a further 5 days before leaf discs were cut from the infiltrated areas (included the injection zone), freeze-dried, weighed and stored at −80°C. Each analyzed sample consisted of discs from four independent infiltrations.

### Total lipid extraction and fatty acid profile analysis

Total lipids were extracted from freeze-dried *N. benthamiana* leaves. Freeze dried leaf tissue was ground to powder in a microcentrifuge tube containing a metallic ball using Reicht tissue lyser (Qiagen) for 3 min. at 20 frequency/s. Chloroform:methanol (2:1, v:v) was added and mixed for a further 3 min. on the tissue lyser before the addition of 1:3 (v:v) of 0.1 M KCl. The sample was then mixed for a further 3 min. before centrifugation (5 min. at 14,000 g), after which the lower lipid phase was collected. The remaining phase was washed once with chloroform, and the lower phase extracted and pooled with the earlier extract. Lipid phase solvent was then evaporated completely using N_2_ gas flow and the lipids resuspended in 5 μL chloroform per mg of original dry leaf weight.

Fatty acid methyl esters (FAMEs) of total lipids were produced by incubating extracted lipid in 1 N methanolic-HCl (Supelco, Bellefonte, PA) at 80°C for 3 h. FAMEs were analyzed by an Agilent 7890A gas chromatograph coupled with flame ionization detector (GC-FID, Agilent Technologies, Palo Alto, CA), on a BPX70 column (30 m, 0.25 mm inner diameter, 0.25 μm film thickness, SGE) essentially as described previously (Zhou et al., 2011), except the column temperature program. The column temperature was programmed as an initial temperature at 100°C holding for 3 min, ramping to 240°C at rate of 7°C/min and holding for 1 min. NuChek GLC-426 was used as the external reference standard. Peaks were integrated with Agilent Technologies ChemStation software [Rev B.04.03 (16)].

### LC-MS analysis

Lipids extracted from 1 mg dry leaf weight were dissolved in 0.2 mL butanol:methanol (1:1, v/v) and analyzed by liquid chromatography-mass spectrometry (LC-MS), based on previously described methods (Petrie et al., 2012). Briefly, an Agilent 1290 series LC and 6490 triple quadrupole LC-MS with Jet Stream ionization. The phosphatidylcholine (PC) and lysophosphatidylcholine (LPC) species were separated on a Poroshell 120 HILIC column (100 × 2.1 mm, 2.7 μm), over a gradient from 95% acetonitrile to 75% acetonitrile with 20 mM ammonium acetate. PC and LPC hydrogen adducts were quantified by the characteristic 184 *m/z* phosphatidyl head group ion under positive ionization mode. The ammonium adducts of monogalactosyl diacylglycerol (MGDG), digalactosyl diacylglycerol (DGDG), diacylglycerol (DAG) and TAG lipid species were analyzed by the neutral loss of singular fatty acids C_12_ to C_18_. Multiple reaction monitoring (MRM) lists were based on the following major fatty acids: C12:0, C14:0, C16:0, C16:3, C18:0, C18:1, C18:2, C18:3, using a collision energy of 28 V. Lipids were chromatographically separated using an Agilent Poroshell column (50 mm × 2.1 mm, 2.7 μm) and a binary gradient with a flow rate of 0.2 mL/min. The mobile phases were: A. 10 mM ammonium formate in H_2_O:acetonitrile: isopropanol (5:45:50, v/v); B. 10 mM ammonium formate in H_2_O:acetonitril: isopropanol (5:20:75, v/v). Individual MRM TAG was identified based on ammoniated precursor ion and product ion from neutral loss.

## Results

### A survey of thioesterase activity in the production of MCFA in leaves

Seven thioesterases from organisms known to produce MCFAs were infiltrated along with the silencing suppressor gene (p19) to determine whether MCFA could be produced in *N. benthamiana* leaf tissue. Leaf spots were harvested and freeze-dried after 5 days, after which the total fatty acid profiles were determined by GC (Table 1). Control leaf was found to contain trace or non-detectable levels of C12:0 and C14:0, whereas C16:0 was present at 14.9 ± 0.6% in total leaf lipids. C12:0 accumulation was observed at low levels with expression of Cocnu-TE3 (1.2 ± 0.1%) and Umbca-TE (1.6 ± 0.1%). Expression of all the thioesterases tested resulted in the accumulation of C14:0 in *N. benthamiana* leaf with Cinca-TE giving the greatest response of 11.3 ± 1.0%. Similarly, all of the thioesterases resulted in increased C16:0 accumulation with the exception of Umbca-TE. The greatest C16:0 accumulation was observed with Cocnu-TE1 expression (35.4 ± 4.7%).

**Table 1:**
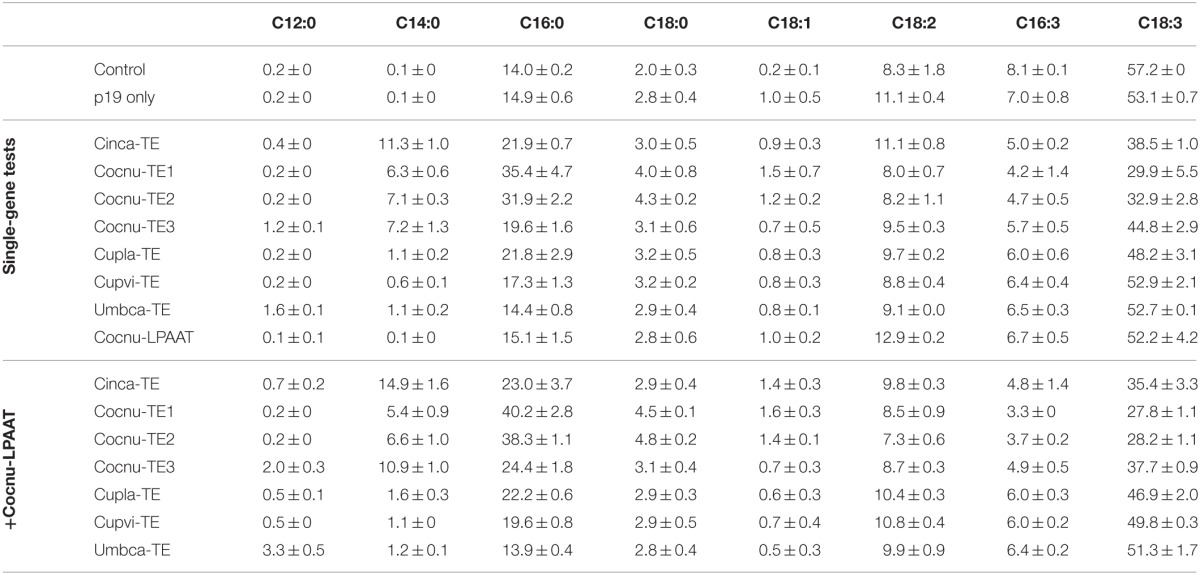
**Leaf total fatty acid methyl ester (FAME) profiles (weight %) of *Nicotiana benthamiana* leaf infiltrated with various thioesterases (TE) and the *Cocos nucifera* lysophosphatidic acid acyltransferase (LPAAT)**.

*“Control” denotes uninfiltrated N. benthamiana leaf whereas “p19 only” contains the viral suppressor gene alone. p19 was included in all samples, errors are standard deviation of triplicate infiltrations, “C16:3” is C16:3^Δ*7,10,13*^ and “C18:3” is C18:3^Δ*9,12,15*^. Gene identities are defined in the manuscript*.

### The co-expression of the coconut LPAAT increases MCFA production in leaf

The *C. nucifera* LPAAT (CnLPAAT) had previously been shown to increase MCFA incorporation on the *sn*-2 position of TAG (Knutzon et al., 1999). This gene was co-infiltrated with the thioesterases described above. The co-infiltration of CnLPAAT and Umbca-TE increased the accumulation of C12:0 to 3.3 ± 0.5% and C14:0 was found to accumulate to 14.9 ± 1.6% in the Cinca-TE + CnLPAAT sample (Table 1). The highest C16:0 levels were observed after co-expression of Cocnu-TE1 and CnLPAAT (40.2 ± 2.8%). Some leaf chlorosis was observed following Cocnu-TE1 infiltration, so Cocnu-TE2 was used for further experiments since it also resulted in high C16:0 accumulation (38.3 ± 1.1%).

### MCFA production in combination with AtWRI1 and AtDGAT1, a high oil leaf background

We were interested in whether the accumulation of MCFA that we observed after expression of thioesterases with CnLPAAT would also be observed in leaves which were co-expressing AtWRI1 and AtDGAT1, a combination that was previously found to result in a 100-fold accumulation of oil in transient *N. benthamiana* leaf expression tests (Vanhercke et al., 2013).

**Figure 1:**
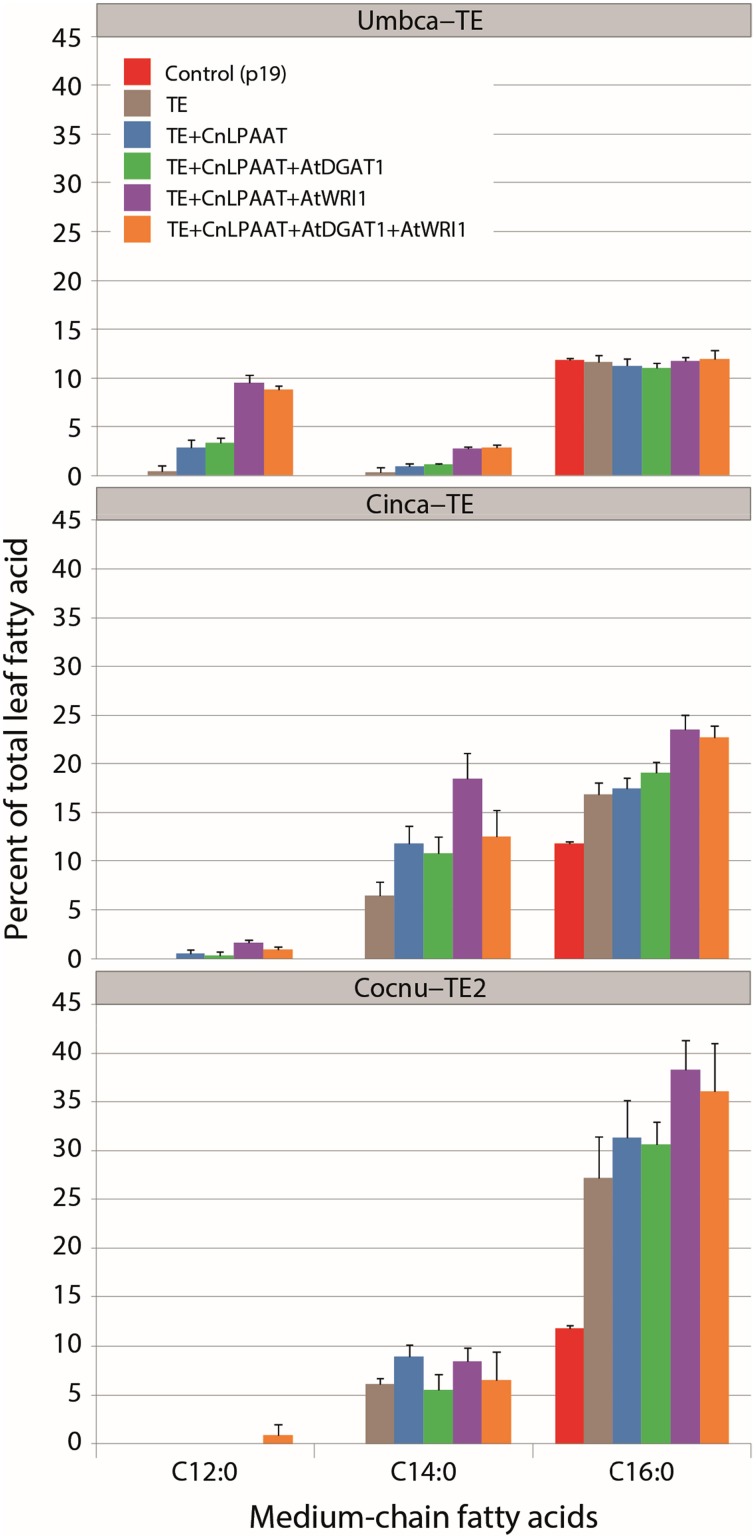
**Total fatty acid methyl ester (FAME) profiles (weight %) illustrating the effect of WRI1+DGAT1-mediated high oil background on MCFA production in *Nicotiana benthamiana* leaf (*n* = 4)**. This subset of the data highlights the accumulation of MCFA (complete dataset can be found in Supplementary Table 1). Highest MCFA production was observed after the addition of Arath-WRI1 and in the absence of Arath-DGAT1. Umbca-TE is *Umbellularia californica* C12:0-ACP thioesterase; Cinca-TE is *Cinnamomum camphora* C14:0-ACP thioesterase; Cocnu-TE2 is *Cocos nucifera* acyl-ACP thioesterase FatB2.

The best performing CnLPAAT plus C12:0-, C14:0-, and C16:0-thioesterase combinations (Umbca-TE, Cinca-TE and Cocnu-TE2 thioesterases, respectively) were infiltrated in combination with AtWRI1, AtDGAT1 and a combination of AtWRI1 +AtDGAT1 (Figure 1). The accumulation of the relevant MCFA (C12:0 for Umbca-TE, C14:0 for Cinca-TE, and C16:0 for Cocnu-TE2) was in each instance most affected by the addition of AtWRI1 to the CnLPAAT + TE combination: C12:0 constituted 9.5 ± 0.9% of total leaf fatty acids in the Umbca-TE + CnLPAAT + AtWRI1 samples, C14:0 constituted 18.5 ± 2.6% in the Cinca-TE + CnLPAAT + AtWRI1 samples and C16:0 constituted 38.3 ± 3.0% in the Cocnu-TE2 + CnLPAAT + AtWRI1 samples. No significant improvement was observed in the accumulation of MCFA following the co-expression of AtDGAT1. Levels of C18:3^Δ9,12,15^ were negatively correlated with MCFA levels (Supplementary Table 1), most notably in the Cocnu-TE2 + CnLPAAT + AtWRI1 sample where the C18:3^Δ9,12,15^ amount nearly halved from 62.2 ± 1.4 to 32.5 ± 3.3%.

### Lipid species analysis by LC-MS

LC-MS was used to gain a better understanding of MCFA accumulation and whether the newly-produced MCFA were accumulating in the neutral, polar or plastidial lipids.

**Figure 2:**
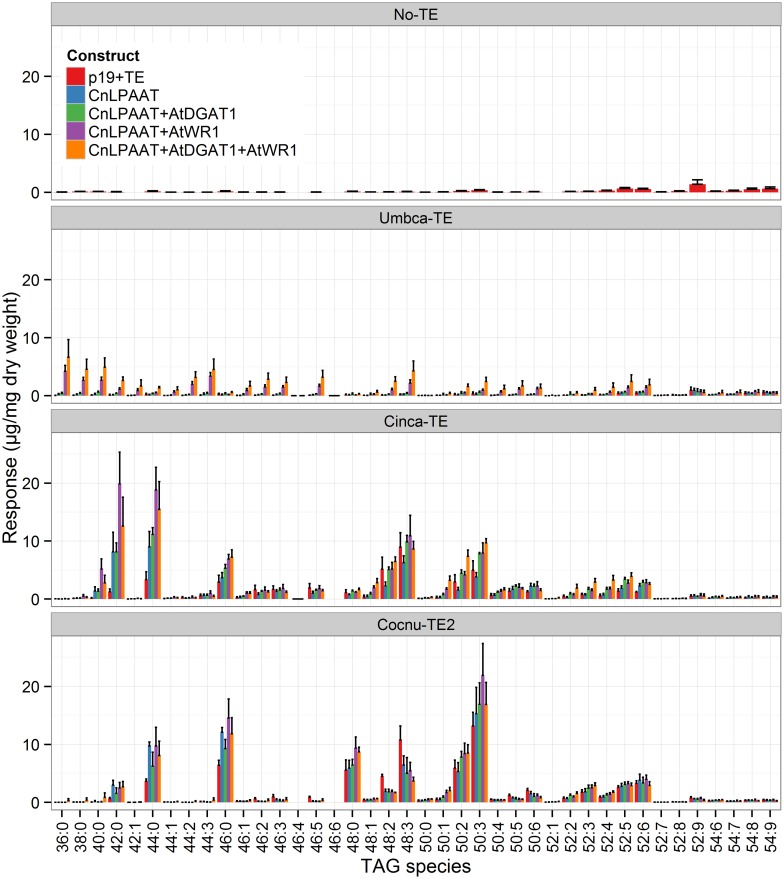
**Full dataset of triacylglycerol (TAG) species for infiltrated *Nicotiana benthamiana* leaf samples (*n* = 4)**. The label “No-TE” represents the *N. benthamiana* control leaf that was infiltrated with p19 only. With the expression of each thioesterase, in combination with CnLPAAT, AtWRI1 and AtDGAT1, significant increases in total TAG levels were observed. The data has been normalized using the internal standard tri-C17:0 TAG (51:0) (y-axis), being presented in units of 1 μg/mg leaf dry weight.

**Figure 3:**
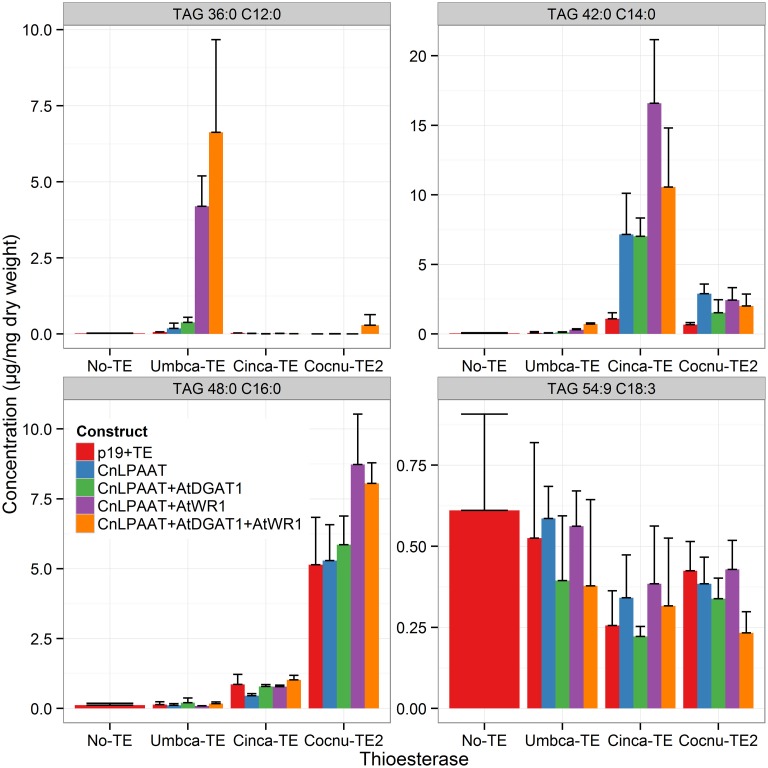
**Important TAG species illustrating the accumulation of MCFA in TAG (*n* = 4)**. The label “No-TE” represents the *Nicotiana benthamiana* control leaf that was infiltrated with p19 only. Following the expression of appropriate thioesterases MCFA TAG species including tri-C12:0 (TAG 36:0), -C14:0 (TAG 42:0) and -C16:0 (TAG 48:0), were observed.

**Figure 4:**
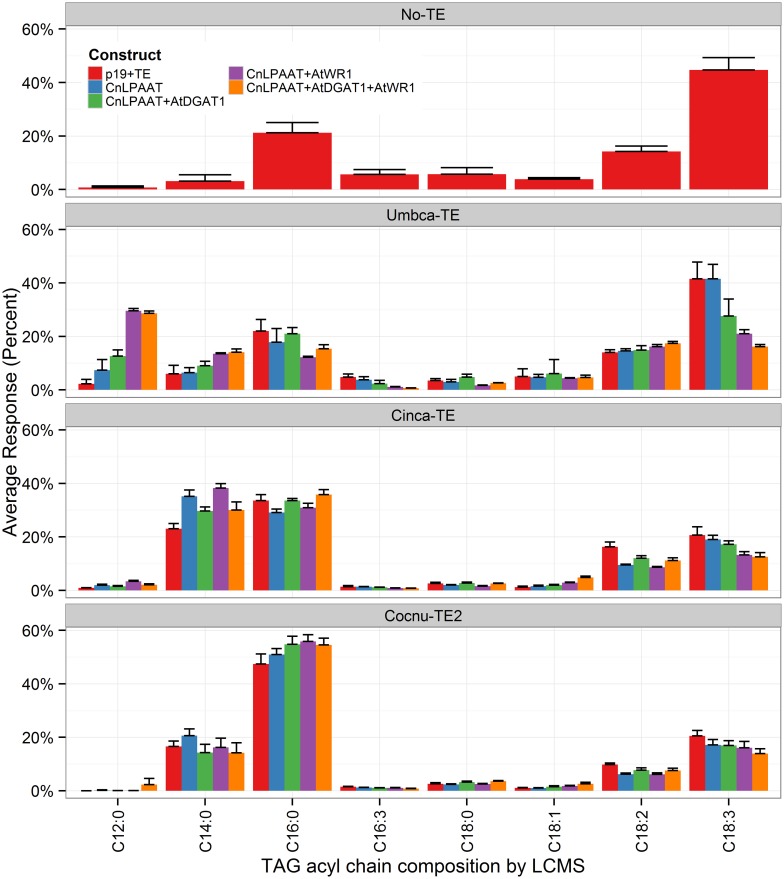
**Acyl chain composition of TAG was analyzed via liquid-chromatography mass-spectrometry (LCMS), for infiltrated *Nicotiana benthamiana* leaf samples (*n* = 4)**. The label “No-TE” represents the *N. benthamiana* control leaf that was infiltrated with p19 only. The data is presented as the percentage composition of each acyl chain from total TAG (y-axis). High levels of MCFA (C12:0, C14:0, and C16:0) were shown to accumulate in TAG.

MCFA was found to accumulate in TAG (Figure 2). The major TAG species were C12:0-containing for the Umbca-TE samples, C14:0-containing for the Cinca-TE samples and C16:0-containing for the Cocnu-TE2 samples. For the Umbca-TE, there was limited accumulation of low molecular weight TAG species (containing C12:0) until coexpressed with CnLPAAT and AtWRI (Umbca-TE only = 0.1 ± 0.1 μg/mg; Umbca-TE + CnLPAAT = 0.6 ± 0.5 μg/mg; Umbca-TE + CnLPAAT + AtWRI1 = 11.8 ± 2.6 μg/mg), with an additive effect when also expressed with AtDGAT1 (Umbca-TE + CnLPAAT + AtWRI1 + AtDGAT1 = 19.8 ± 8.3 μg/mg). Following the over-expression of thioesterases, the accumulation of corresponding tri-MCFA TAG species was observed (Figure 3). These tri-MCFA species were only found to accumulate upon the co-expression of the CnLPAAT, with the MCFA being incorporated into the sn-2 position of TAG (Knutzon et al., 1999). This observation was particularly noted in the cases of tri-C12:0 (TAG 36:0) with Umbca-TE (TE only <0.1 μg/mg; Umbca-TE + CnLPAAT = 0.2 ± 0.2 μg/mg; Umbca-TE + CnLPAAT + AtWRI1 = 4.2 ± 1.0 μg/mg) and tri-C14:0 (TAG 42:0) with Cinca-TE (TE only = 1.1 ± 0.4 μg/mg; Cinca-TE + CnLPAAT, 7.2 ± 3.0 μg/mg; Cinca-TE + CnLPAAT + AtWRI1 = 16.6 ± 4.6 μg/mg). It was also observed that the addition of AtWRI1 further increased the abundance of these tri-MCFA TAG species. However, it appeared that C16:0 acyl chains appear to be incorporated more easily into TAG species, with the production of tri-C16:0 (TAG 48:0) being observed with over-expression of Cocnu-TE2 only (Control-p19 = 0.1 ± 0.01 μg/mg; Cocnu-TE2 alone = 5.1 ± 1.7 μg/mg), most likely through the expression of native acyltransferases. Through investigation of the acyl chain composition of TAG it was observed that a significant proportion of TAG contained the newly-produced MCFA (Figure 4) (Umbca-TE + CnLPAAT + AtWRI1 = 29.6 ± 0.9% C12:0; Cinca-TE + CnLPAAT + AtWRI1 = 38.2 ± 1.7% C14:0; Cocnu-TE2 + CnLPAAT + AtWRI1 = 55.8 ± 2.6% C16:0).

**Figure 5:**
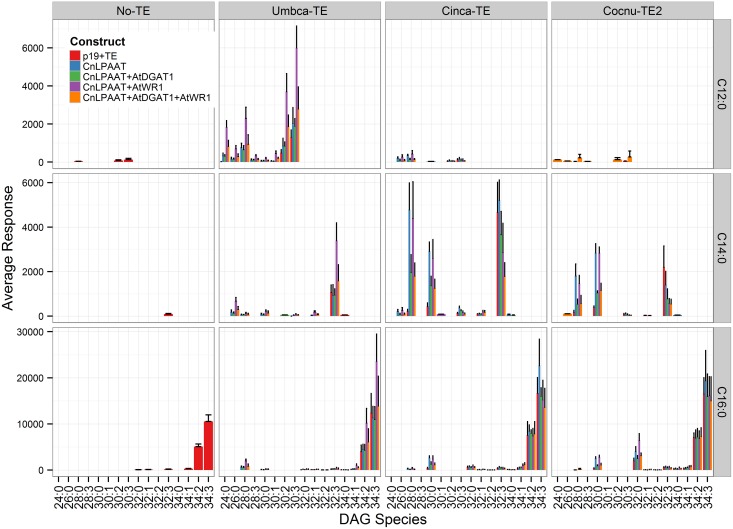
**Full dataset of diaclyglycerol (DAG) species for infiltrated *Nicotiana benthamiana* leaf samples (*n* = 4)**. The label “No-TE” represents the *N. benthamiana* control leaf that was infiltrated with p19 only. Each row represents DAG species containing at least one of the corresponding acyl chains (labeled on right). The data is represented according to LC-MS response (y-axis).

Similar analysis was performed for the DAG class (Figure 5). With thioesterase expression alone, C12:0 and C14:0 species were predominantly found with C18:3 acyl chains (DAG 30:3 C12:0/C18:3, DAG 32:3 C14:0/C18:3). Co-expression of LPAAT with the C12:0 and C14:0 thioesterases significantly increased di-MCFA DAG species accumulation. DAG 24:0 (C12:0/C12:0) was not detected after expression of Umbca-TE alone, while in the presence of the LPAAT it was detected at 0.4 ± 0.3% (expressed as percent of total DAG species). Total acyl C12:0 after Umbca-TE expression increased from 2.2 ± 0.2 to 4.9 ± 0.7% with the addition of LPAAT. DAG 28:0 (C14:0/C14:0) was detected at 0.2 ± 0.1% with expression of Cinca-TE and 4.0 ± 1.0% with LPAAT co-expression. Total C14:0 acyl chain in Cinca-TE expression increased from 5.5 ± 1.0 to 10.0 ± 2.0% with the addition of LPAAT. The expression of Cocnu-TE2 alone resulted in the production of di-MCFA DAG 32:0 (C16:0/C16:0), suggesting that lipid handling pathways in *N. benthamiana* leaf have lower specificity for chains shorter than C16:0. Co-expression of the AtDGAT1 consistently decreased DAG species with MCFA indicating that further increases in MCFA accumulation may require a DGAT with preference for MCFA substrates.

**Figure 6:**
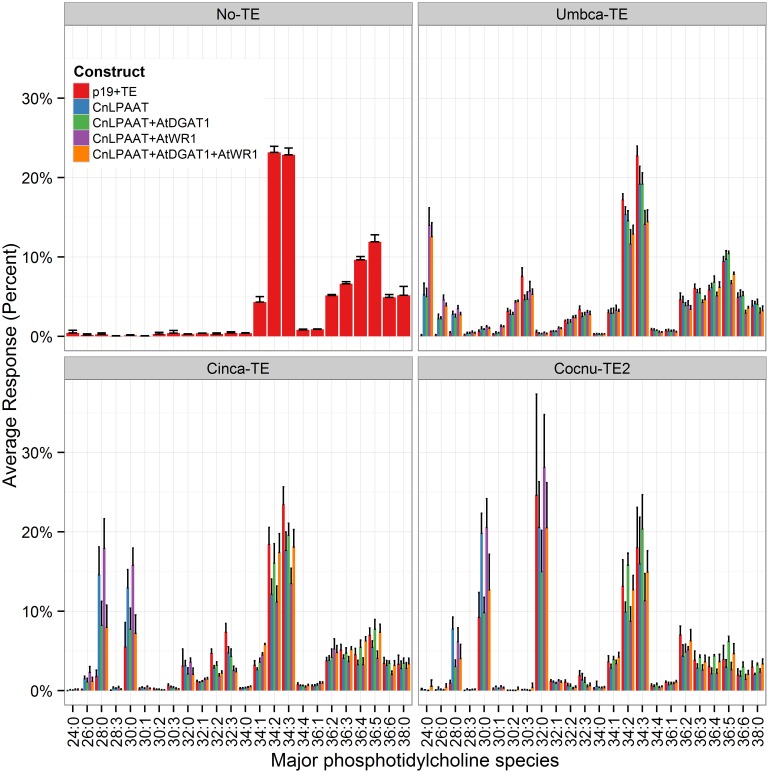
**Major phosphatidylcholine (PC) species in *Nicotiana benthamiana* control leaf (top left) or leaf transiently expressing a thioesterase either alone or in combination with the *Cocos nucifera* LPAAT + *Arabidopsis thaliana* WRI1 + *A. thaliana* DGAT1 (*n* = 4)**. The label “No-TE” represents the *N. benthamiana* control leaf that was infiltrated with p19 only. The percentage of each PC species for each sample is shown on the y-axis.

The presence of MCFA in the polar lipid PC was also examined (Figure 6). All samples with thioesterase expression were found to contain C12:0, C14:0, or C16:0 in PC. The addition of AtWRI1 resulted in the increased accumulation of di-MCFA species in the PC pool, particularly with the expression of Umbca-TE (PC 24:0 (di-C12:0) Umbca-TE + CnLPAAT = 5.3 ± 1.4%; Umbca-TE + CnLPAAT + AtWRI = 14.0 ± 2.2%) and Cinca-TE (PC 28:0 (di-C14:0) Cinca-TE + CnLPAAT = 14.6 ± 3.5%; Cinca-TE + CnLPAAT + AtWRI = 17.9 ± 3.7%). Similar acyl chain assembly was observed between DAG and PC lipid pools. However, there was a consistent greater percentage increase in the accumulation of MCFA PC species than in the DAG pool. The results were particularly striking with Cocnu-TE2 expression with PC 32:0 representing between 15 and 30% of the PC pool depending on the expression of lipid handling genes, with the highest levels observed with the expression of Cocnu-TE2 + LPAAT + WRI1 (28 ± 7%). In contrast the DAG 32:0 (di-C16:0) was 2.2 ± 0.1% of the total DAG pool when expressed with Cocnu-TE2 alone. Similar results were also observed with investigations involving the expression of Umbca-TE (Umbca-TE + LPAAT + AtWRI, DAG 24:0 (di-C12:0) = 1.1 ± 0.1%, PC 24:0 = 14 ± 2%), and Cinca-TE (Cinca-TE + LPAAT + AtWRI DAG 28:0 (di-C14:0) = 4.4 ± 0.9, PC 28:0 17 ± 3%).

**Figure 7:**
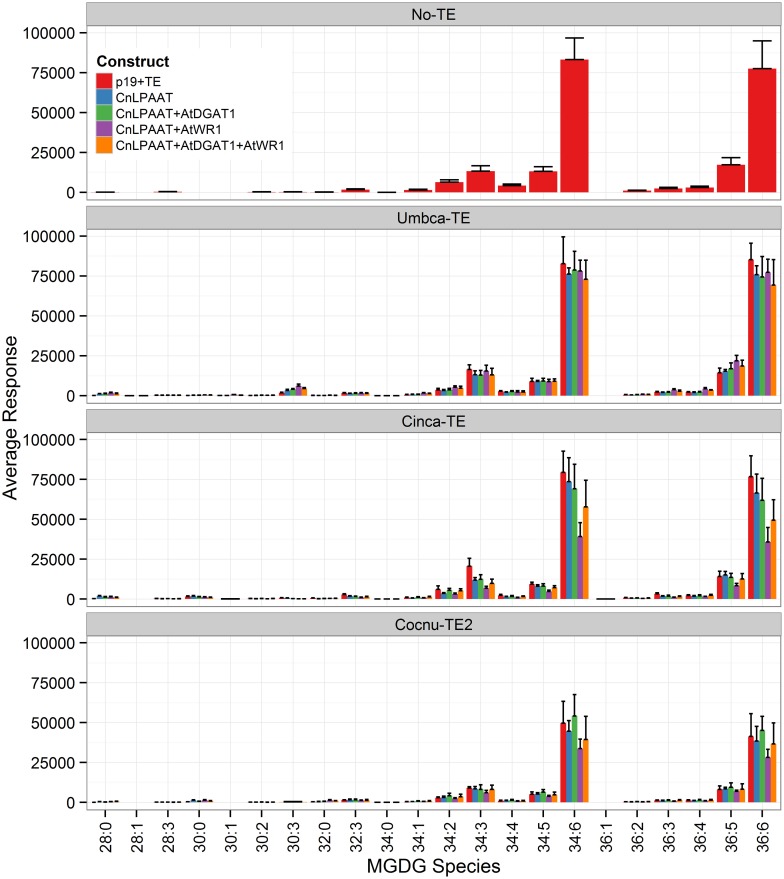
**Full dataset of monogalactosyl diaclyglycerol (MGDG) species for infiltrated *Nicotiana benthamiana* leaf samples (*n* = 4)**. The label “No-TE” represents the *N. benthamiana* control leaf that was infiltrated with p19 only. The data is represented according to LC-MS response (y-axis).

**Figure 8:**
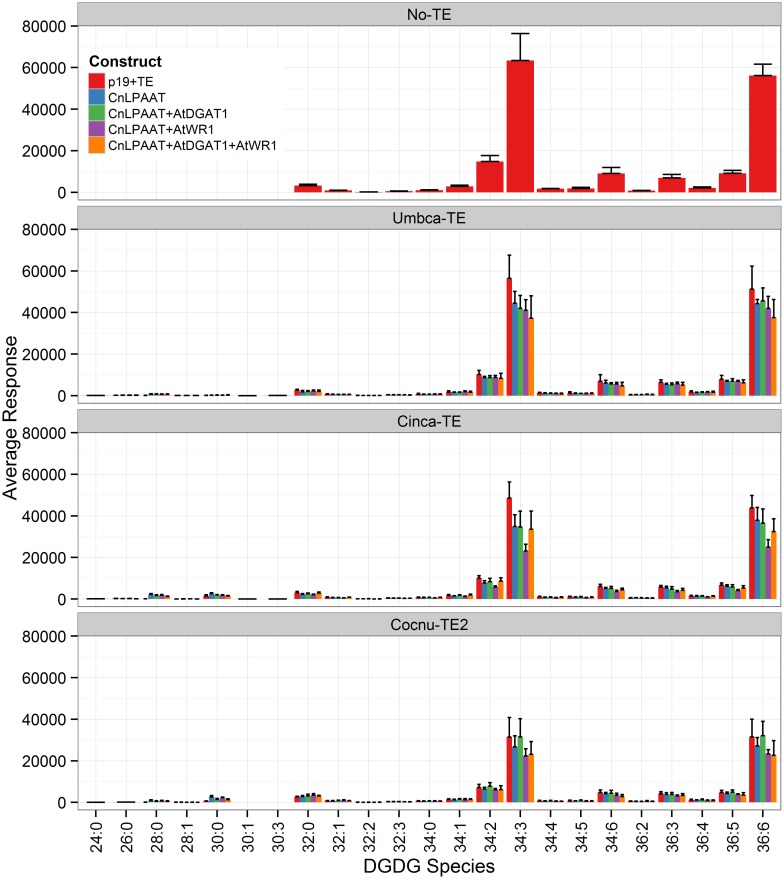
**Full dataset of digalactosyl diaclyglycerol (DGDG) species for infiltrated *Nicotiana benthamiana* leaf samples (*n* = 4)**. The label “No-TE” represents the *N. benthamiana* control leaf that was infiltrated with p19 only. The data is represented according to LC-MS response (y-axis).

The plastidial galactolipids monogalactosyl diacylglycerol (MGDG) (Figure 7) and digalactosyl diacylglycerol (DGDG) (Figure 8) contained only low levels of C12:0 and C14:0 and reduced levels of C16:0 relative to the p19 control leaf. The major C12:0-containing MGDG species in the Umbca-TE samples was 30:3 indicating that C18:3 and C12:0 were co-located on the monogalactosyl backbone. Although this demonstrates that MCFAs are being assembled into MGDG, the 30:3 species represents only 1.3 ± 0.3% of the total MGDG pool, with the co-expression of AtWRI1. The other main C12:0-containing MGDG species was 28:0, indicating that the second fatty acid was C16:0. The major C14:0-containing MGDG species in the Cinca-TE samples were 28:0 and 30:0, indicating that a significant proportion of the C14:0 in MGDG was either di-C14:0 or co-located with C16:0. The C12:0-containing and C14:0-containing MGDG species were not detected in the p19 control sample. In contrast, C16:0-containing MGDG species tended to be reduced in the Cocnu-TE2 samples. The major MGDG species in the wildtype samples (MGDG 34:6 C16:3/C18:3, and MGDG 36:6 C18:3/C18:3) all tended to be reduced by the expression of the transgenes. This reduction was greatest in the presence of the WRI+DGAT combination. Only trace levels of C12:0-containing DGDG species were observed in the Umbca-TE samples. The major C14:0-containing DGDG species observed in the Cinca-TE samples were 28:0 and 30:0, both of which were absent in the control. These species were also observed at elevated levels in the Cocnu-TE2 samples but only at trace levels in the Umbca-TE samples. The major DGDG species in the wildtype samples (DGDG 34:3 C16:0/C18:3 and DGDG 36:6 C18:3/C18:3) all tended to be reduced by the expression of the transgenes. This reduction was greatest in the presence of AtWRI.

**Figure 9:**
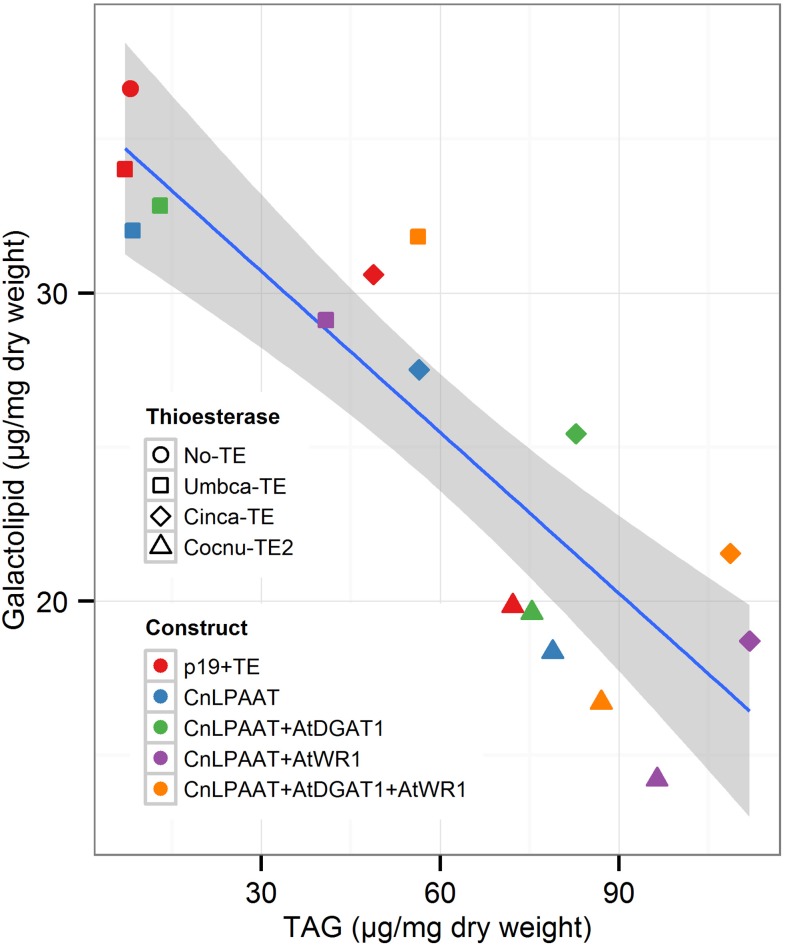
**Correlation between normalized levels of TAG (x-axis) and galactolipids (y-axis), from *Nicotiana benthamiana* leaf infiltration samples**. The blue line represents the linear model, with 95% confidence. Each data point represents the mean value (*n* = 4) for each treatment. The shape of each data point is representative of the thioesterase used. The color of each data point represents the corresponding gene combinations.

We were also interested in the correlation between the proportions of plastidial galactolipids and TAG (Figure 9). The control samples (p19 only, red circles in Figure 9) show that galactolipids are typically much more abundant than TAG (p19 only total TAG, 8.0 ± 2.4 μg/mg dry weight; total galactolipid, 36.6 ± 3.7 μg/mg dry weight) (Supplementary Table 2). In the case of the *U. californica* lauroyl-ACP thioesterase, this is not greatly changed by the co-expression of CnLPAAT + Umbca-TE (red vs. blue squares) (8.4 ± 1.9 μg/mg dry weight) or the further addition of AtDGAT1 (green square) (13.0 ± 3.7 μg/mg dry weight). The relative proportion of TAG is, however, seen to increase with the further addition of AtWRI1 (purple square, orange square) (with CnLPAAT+AtWRI1, 40.8 ± 8.0 μg/mg dry weight; with CnLPAAT + AtWRI1 + AtDGAT1, 56.3 ± 3.2 μg/mg dry weight). This is in contrast with both the results observed for the *C. camphora* C14:0-ACP and *C. nucifera* C16:0-ACP thioesterases in which the co-expression of CnLPAAT + Cinca-TE (blue diamond) or Cocnu-TE2 (blue triangle) increased the relative proportion of TAG to galactolipids even without the addition of the AtWRI1 or AtDGAT1. TAG was found to be most abundant relative to galactolipids after the co-expression of AtWRI1 + CnLPAAT + Cinca-TE (111.9 ± 23.4 μg/mg dry weight) or Cocnu-TE2 (96.4 ± 10.4 μg/mg dry weight). Complete data for the correlation between TAG and galactolipids is found in Supplementary Table 2.

## Discussion

Earlier work described the constitutive expression in *B. napus* of the *U. californica* lauroyl-ACP thioesterase (Eccleston et al., 1996), the Umbca-TE gene used in this study. This resulted in the accumulation of significant levels of C12:0 in seed oil but only trace levels in leaf despite high transcript abundance and lauroyl-ACP thioesterase activity. In contrast, C12:0 synthesis was observed during *in vitro* feeding of [1-^14^C] acetate to isolated leaf plastids from these transgenic events. Our study showed similar results in that accumulation of C12:0 was found to be very low (1.6% of total leaf fatty acids) after expression of Umbca-TE alone (Table 1). The co-expression of the CnLPAAT, reported to have a preference for MCFA (Davies et al., 1995), with the individual thioesterases increased the accumulation of C12:0, C14:0, and C16:0, all of which are found in *C. nucifera* oil (Laureles et al., 2002). This result indicated that the native *N. benthamiana* LPAAT was either not highly expressed in leaf or did not have high specificity for C12:0, C14:0, and C16:0 substrates.

The co-expression of the AtWRI1 with the CnLPAAT and thioesterases had an additional positive effect on C12:0 and C14:0 accumulation in leaf tissue. The further addition of AtDGAT1 to the thioesterase + CnLPAAT + AtWRI1 combinations did not further increase MCFA accumulation. In fact, in most cases the addition of AtDGAT1 decreased MCFA accumulation, suggesting that this enzyme may not have good activity on MCFA substrates (Aymé et al., 2014). However, it is important to note that tri-C12:0, -C14:0, and -C16:0 TAG species were detected, indicating that the CnLPAAT and native *N. benthamiana* lipid assembly enzymes were able to utilize MCFA substrates to some extent. In contrast with the C12:0- and C14:0-ACP thioesterases, it was consistently noted that the C16:0-ACP thioesterase samples were not as strongly affected by the co-expression of At WRI1. The strongest WRI1-effect was observed with the C12:0-ACP thioesterase, with the C14:0-ACP thioesterase having an intermediate response to the addition of AtWRI1. We speculate that this could be due to relatively rapid KASI-mediated condensation of C4:0 to C14:0 in the plastid with C12:0 being relatively inaccessible to a C12:0-ACP thioesterase for cleavage from ACP.

Lipid species analysis by LC-MS was also informative although it is important to understand that the transient nature of the assay means that results are not necessarily representative of stably transformed plants. The apparent reductions in levels of plastidial lipids MGDG and DGDG could have been due to increased export of fatty acids from the plastid before they could be incorporated into plastidial glycerolipids by the native *N. benthamiana* plastidial GPAT. The presence of MCFA in MGDG indicated that the plastidial GPAT was capable of accessing MCFA-ACP that remained in the plastid as substrate. Alternatively, the increased extra-plastidial MCFA could have been imported back into the plastid from the ER following thioesterase export. It is worthwhile noting the substantial accumulation of MCFA in PC which could be occurring via the fatty acids first being incorporated into *de novo* DAG, which is then converted to PC via the action of choline phosphotransferase (CPT) or phosphatidylcholine diacylglycerol phosphotransferase (PDCT) (Bates et al., 2013). It is also possible that MCFA could be incorporated in PC via direct acylation of *sn*-1 LPC via the action of lysophosphatidylcholine acyltransferase (LPCAT) (Lager et al., 2013). The presence of di-C12:0 PC and di-C14:0 PC does suggest that the above two routes of MCFA incorporation into PC might be operating under the transient leaf assay settings.

It remains to be seen how stable transgenic plants incorporating MCFA in PC fare, as the presence of MCFA could alter membrane fluidity significantly. Some chlorosis was observed in infiltrated leaves during the transient assay, similar to the phenotype observed following monoacylglycerol acyltransferase (MGAT1) transient expression (Divi et al., 2014), possibly as a result of MCFA accumulation in PC. The reduction in total galactolipids, MGDG (Figure 7) and DGDG (Figure 8) was also interesting since these lipid pools are essential to the efficiency of photosynthetic light reactions (Dörmann, 2013). Further investigations into sequestering MCFA into TAG are required. It is also possible that in a stable setting MCFA might be actively edited out of PC by the very active unusual fatty acyl editing mechanisms present in plant cells and sequestered in TAG (Millar et al., 2000; Lager et al., 2013).

Eccleston et al. (1996) hypothesized that the lack of MCFA accumulation observed in *B. napus* leaf was due to the breakdown of the unusual fatty acids by β-oxidation, a concept supported by an observed increase in isocitrate lyase activity. If WRI1 expression does not adequately down-regulate such breakdown pathways in a leaf context it is possible that the co-expression of WRI1 simply increased fatty acid synthesis with a corresponding increase in plastidial export by the transgenic thioesterases. It was interesting to observe the inverse relationship between TAG and galactolipids which occurred after co-expression of the *A. thaliana* acyltransferases with the *U. californica* lauroyl-ACP thioesterase and CnLPAAT, or even in their absence in the case of the *C. camphora* C14:0-ACP and *C. nucifera* C16:0-ACP thioesterases.

Much of the C12:0, C14:0, and C16:0 were found to accumulate in TAG. This was important since the production of industrially-relevant levels of leaf TAG have already been demonstrated in tobacco (Vanhercke et al., 2014a). Our study offers a potential extension of this result by demonstrating that it is possible to tailor leaf oil profiles to produce industrially-relevant fatty acids in leaves with elevated energy content.

## Conclusion

The experiments performed used the rapid *N. benthamiana* assay to demonstrate the activity of a series of thioesterases in leaf tissue. The study demonstrated that the over-expression of AtWRI1and CnLPAAT along with the thioesterases significantly increased the accumulation of C12:0 and C14:0 in leaf. Importantly, newly-produced fatty acids were effectively incorporated into leaf TAG. Collectively, these results indicate that high oil-containing plant leaves could be a good host for MCFA production.

### Conflict of interest statement

The authors declare that the research was conducted in the absence of any commercial or financial relationships that could be construed as a potential conflict of interest.
