# Supplementary material for: Metabolic engineering of medium-chain fatty acid biosynthesis in Nicotiana benthamiana plant leaf lipids
Source: Front Plant Sci. 2015 Mar 24;6:164. doi: 10.3389/fpls.2015.00164 (PMC4371700; doi:10.3389/fpls.2015.00164)
Supplement: Supplementary file 1 [file Table1.DOCX]

**Supplementary Table 1. Full WRI-DGAT Assay profile**

Total fatty acid methyl ester (FAME) profiles (weight %) illustrating the effect of WRI1+DGAT1-mediated high oil background on MCFA production in *Nicotiana benthamiana* leaf (n=4).

|  | **Treatment (Genes)** | **C12:0** | **C14:0** | **C16:0** | **C16:3** | **C18:0** | **C18:1** | **C18:2** | **C18:3** |
| --- | --- | --- | --- | --- | --- | --- | --- | --- | --- |
|  |  |  |  |  |  |  |  |  |  |
|  | **P19 only** | 0.0±0.0 | 0.0±0.0 | 11.8±0.2 | 8.6±0.6 | 1.8±0.1 | 1.2±0.2 | 9.9±0.3 | 62.2±1.4 |
|  |  |  |  |  |  |  |  |  |  |
| **+UmbcaTE** | **P19+UmbcaTE** | 0.5±0.6 | 0.4±0.4 | 11.6±0.7 | 8.5±0.5 | 1.8±0.2 | 0.5±0.3 | 8.0±1.0 | 65.1±0.9 |
|  | **P19+UmbcaTE+CnLPAAT** | 2.9±0.8 | 1.0±0.3 | 11.2±0.8 | 7.8±0.4 | 1.8±0.1 | 0.8±0.2 | 8.6±0.3 | 61.5±1.1 |
|  | **P19+UmbcaTE+CnLPAAT+DGAT1** | 3.3±0.6 | 1.2±0.0 | 11.1±0.4 | 7.7±0.5 | 1.8±0.1 | 0.9±0.2 | 9.4±0.2 | 60.7±0.7 |
|  | **P19+UmbcaTE+CnLPAAT+WRI1** | 9.5±0.9 | 2.8±0.2 | 11.7±0.5 | 6.3±0.3 | 1.7±0.1 | 1.9±0.3 | 10.4±0.4 | 52.1±1.4 |
|  | **P19+UmbcaTE+CnLPAAT+DGAT1+WRI1** | 8.8±0.4 | 2.8±0.3 | 11.9±0.9 | 6.4±0.3 | 1.8±0.1 | 2.0±0.3 | 11.2±0.5 | 51.1±0.6 |
|  |  |  |  |  |  |  |  |  |  |
| **+CincaTE** | **P19+CincaTE** | 0.0±0.0 | 6.5±1.4 | 16.9±1.2 | 7.2±0.8 | 2.1±0.2 | 0.7±0.3 | 9.3±1.4 | 53.2±3.1 |
|  | **P19+CincaTE+CnLPAAT** | 0.5±0.4 | 11.8±1.8 | 17.5±1.1 | 6.2±0.2 | 2.3±0.1 | 0.9±0.2 | 8.4±0.6 | 47.7±3.0 |
|  | **P19+CincaTE+CnLPAAT+DGAT1** | 0.3±0.4 | 10.8±1.7 | 19.0±1.1 | 6.0±0.4 | 2.3±0.2 | 1.3±0.2 | 10.2±0.8 | 45.3±2.1 |
|  | **P19+CincaTE+CnLPAAT+WRI1** | 1.6±0.3 | 18.5±2.6 | 23.5±1.5 | 4.4±0.4 | 2.3±0.1 | 1.9±0.1 | 8.1±0.4 | 35.1±3.7 |
|  | **P19+CincaTE+CnLPAAT+DGAT1+WRI1** | 0.9±0.3 | 12.6±2.7 | 22.7±1.3 | 5.0±0.5 | 2.4±0.1 | 2.7±0.5 | 10.3±0.4 | 38.6±4.3 |
|  |  |  |  |  |  |  |  |  |  |
| **+CocnuTE2** | **P19+CocnuTE2** | 0.0±0.0 | 6.2±0.6 | 27.2±4.2 | 6.0±0.7 | 2.7±0.4 | 0.8±0.2 | 7.8±0.6 | 43.7±5.1 |
|  | **P19+CocnuTE2+CnLPAAT** | 0.0±0.0 | 8.9±1.2 | 31.3±3.9 | 5.2±0.6 | 2.7±0.2 | 0.7±0.5 | 6.9±0.3 | 39.2±4.9 |
|  | **P19+CocnuTE2+CnLPAAT+DGAT1** | 0.0±0.0 | 5.5±1.7 | 30.7±2.3 | 5.4±0.2 | 2.9±0.2 | 1.1±0.2 | 8.6±0.3 | 40.9±3.1 |
|  | **P19+CocnuTE2+CnLPAAT+WRI1** | 0.0±0.0 | 8.4±1.5 | 38.3±3.0 | 4.1±0.4 | 2.9±0.3 | 1.1±0.2 | 6.7±0.3 | 32.5±3.3 |
|  | **P19+CocnuTE2+CnLPAAT+DGAT1+WRI1** | 0.9±1.1 | 6.5±3.0 | 36.0±4.9 | 4.3±0.9 | 3.2±0.2 | 1.5±0.3 | 8.1±0.7 | 33.6±7.0 |
