# Supplementary material for: Metabolic engineering of medium-chain fatty acid biosynthesis in Nicotiana benthamiana plant leaf lipids
Source: Front Plant Sci. 2015 Mar 24;6:164. doi: 10.3389/fpls.2015.00164 (PMC4371700; doi:10.3389/fpls.2015.00164)
Supplement: Supplementary file 2 [file Table2.DOCX]

**Supplementary Table 2. Data for TAG vs Galactolipids Correlation**

Numerical data for correlation between normalised levels of triacylglycerol (TAG) and galactolipids, from *Nicotiana benthamiana* leaf infiltration samples (n=4).

|  | **Treatment (Genes)** | **TAG** | | **Galactolipids** | |
| --- | --- | --- | --- | --- | --- |
|  |  | **Mean** | **St.Dev** | **Mean** | **St.Dev** |
|  | **P19 only** | 8.0 | 2.4 | 36.6 | 3.7 |
|  |  |  |  |  |  |
| **+Umbca-TE** | **P19+UmbcaTE** | 7.1 | 3.3 | 34.0 | 1.5 |
|  | **P19+UmbcaTE+CnLPAAT** | 8.4 | 1.9 | 32.0 | 2.3 |
|  | **P19+UmbcaTE+CnLPAAT+DGAT1** | 13.0 | 3.7 | 32.8 | 2.8 |
|  | **P19+UmbcaTE+CnLPAAT+WRI1** | 40.8 | 8.0 | 29.1 | 1.8 |
|  | **P19+UmbcaTE+CnLPAAT+DGAT1+WRI1** | 56.3 | 3.2 | 31.8 | 3.9 |
|  |  |  |  |  |  |
| **+Cinca-TE** | **P19+CincaTE** | 48.8 | 14.1 | 30.6 | 1.7 |
|  | **P19+CincaTE+CnLPAAT** | 56.5 | 15.1 | 27.5 | 1.3 |
|  | **P19+CincaTE+CnLPAAT+DGAT1** | 82.8 | 5.2 | 25.4 | 2.5 |
|  | **P19+CincaTE+CnLPAAT+WRI1** | 111.9 | 23.4 | 18.7 | 3.2 |
|  | **P19+CincaTE+CnLPAAT+DGAT1+WRI1** | 108.7 | 20.7 | 21.5 | 3.9 |
|  |  |  |  |  |  |
| **+Cocnu-TE2** | **P19+CocnuTE2** | 72.1 | 5.8 | 19.8 | 3.3 |
|  | **P19+CocnuTE2+CnLPAAT** | 78.9 | 10.5 | 18.3 | 5.2 |
|  | **P19+CocnuTE2+CnLPAAT+DGAT1** | 75.4 | 13.1 | 19.6 | 4.7 |
|  | **P19+CocnuTE2+CnLPAAT+WRI1** | 96.4 | 10.4 | 14.2 | 2.1 |
|  | **P19+CocnuTE2+CnLPAAT+DGAT1+WRI1** | 87.0 | 6.8 | 16.7 | 5.3 |

The data has been normalized using the internal standard tri-C17:0 TAG (51:0), being presented in units of 1µg/mg leaf dry weight. The mean represents the average value for each treatment group (n=4). The standard deviation (St.Dev.) highlights the variation within each treatment group.
